# A curated collection of *Klebsiella* metabolic models reveals variable substrate usage and gene essentiality

**DOI:** 10.1101/gr.276289.121

**Published:** 2022-05

**Authors:** Jane Hawkey, Ben Vezina, Jonathan M. Monk, Louise M. Judd, Taylor Harshegyi, Sebastián López-Fernández, Carla Rodrigues, Sylvain Brisse, Kathryn E. Holt, Kelly L. Wyres

**Affiliations:** 1 Department of Infectious Diseases, Central Clinical School, Monash University, Melbourne, Victoria 3004, Australia;; 2 Department of Bioengineering, University of California, San Diego, San Diego, California 92093, USA;; 3 Institut Pasteur, Université de Paris, Biodiversity and Epidemiology of Bacterial Pathogens, 75015 Paris, France;; 4 Department of Infection Biology, London School of Hygiene and Tropical Medicine, London WC1E 7HT, United Kingdom

## Abstract

The *Klebsiella pneumoniae* species complex (KpSC) is a set of seven *Klebsiella* taxa that are found in a variety of niches and are an important cause of opportunistic health care–associated infections in humans. Because of increasing rates of multi-drug resistance within the KpSC, there is a growing interest in better understanding the biology and metabolism of these organisms to inform novel control strategies. We collated 37 sequenced KpSC isolates isolated from a variety of niches, representing all seven taxa. We generated strain-specific genome-scale metabolic models (GEMs) for all 37 isolates and simulated growth phenotypes on 511 distinct carbon, nitrogen, sulfur, and phosphorus substrates. Models were curated and their accuracy was assessed using matched phenotypic growth data for 94 substrates (median accuracy of 96%). We explored species-specific growth capabilities and examined the impact of all possible single gene deletions using growth simulations in 145 core carbon substrates. These analyses revealed multiple strain-specific differences, within and between species, and highlight the importance of selecting a diverse range of strains when exploring KpSC metabolism. This diverse set of highly accurate GEMs could be used to inform novel drug design, enhance genomic analyses, and identify novel virulence and resistance determinants. We envisage that these 37 curated strain-specific GEMs, covering all seven taxa of the KpSC, provide a valuable resource to the *Klebsiella* research community.

*Klebsiella pneumoniae* is a ubiquitous bacterium that inhabits a variety of host- and non-host-associated environments and is a major cause of human disease. It is an opportunistic pathogen and a significant contributor to the spread of antimicrobial resistance globally (Pendleton et al. 2013; Navon-Venezia et al. 2017; Thorpe et al. 2021). Multi-drug-resistant *K. pneumoniae* with resistance to the carbapenems (the “drugs of last resort”) cause infections that are extremely difficult to treat and are considered an urgent public health threat (Pendleton et al. 2013). Understanding the biology and ecological behavior of these organisms is essential to inform novel control strategies.

The last 6–7 yr have seen an explosion of *K. pneumoniae* comparative genomics studies, revealing numerous insights into its epidemiology, evolution, pathogenicity, and drug resistance, and informing a genomic framework that facilitates surveillance and knowledge generation (for a recent summary, see Wyres et al. 2020). It is now clear that isolates identified as *K. pneumoniae* through standard microbiological identification techniques actually comprise seven distinct closely related taxa known as the *K. pneumoniae* species complex (KpSC): *K. pneumoniae sensu stricto*, *Klebsiella variicola* subsp. *variicola*, *K. variicola* subsp. *tropica*, *Klebsiella quasipneumoniae* subsp. *quasipneumoniae*, *K. quasipneumoniae* subsp. *similipneumoniae*, *Klebsiella quasivariicola*, and *Klebsiella africana* (Gorrie et al. 2017; Long et al. 2017; Rodrigues et al. 2019; Wyres et al. 2020). *K. pneumoniae sensu stricto* accounts for the majority of human infections and is therefore the most well-studied of these organisms.

Each individual *K. pneumoniae* genome encodes between 5000 and 5500 genes; approximately 2000 are conserved among all members of the species (core genes), and the remainder vary between individuals (accessory genes) (Holt et al. 2015). The total sum of all core and accessory genes is estimated to exceed 100,000 protein-coding sequences that can be assigned to various functional categories, many of which are not well-characterized. For example, the diversity, mechanism, and phenotypic impact of antimicrobial resistance genes, accounting for 1% of the total gene pool, is well understood. In contrast the functional implications of metabolic genes, which account for the largest single fraction of the gene pool (37%) (Holt et al. 2015), are relatively poorly understood. The sheer number of genes in this category suggests that substantial metabolic variability exists within the KpSC, a hypothesis supported by two studies that have generated growth phenotypes for multiple isolates (Brisse et al. 2009; Blin et al. 2017). However, these data are limited by the number and variety of substrates tested and it is difficult to consolidate the genotype data in the context of these phenotypes. Moreover, these phenotyping methods are slow, expensive, and nonscalable across large numbers of isolates.

Genome-scale metabolic modeling represents a powerful approach to bridge the gap between genotypes and phenotypes. Drawing on the accumulated biochemical knowledge base, it is possible to infer the metabolic network of an individual organism from its genome sequence and subsequently apply in silico modeling approaches to predict its metabolic capabilities (growth phenotypes) (Thiele and Palsson 2010; O'Brien et al. 2015). Such models allow exploration of metabolic diversity (Monk et al. 2013; Bosi et al. 2016; Seif et al. 2018), prediction of the impact of gene deletions or the response to drug exposure (Tong et al. 2020), identification of novel virulence factors or drug targets (Bartell et al. 2017; Ramos et al. 2018; Zhu et al. 2018), and optimization for the production of industrially relevant compounds (Jung et al. 2015; Li et al. 2016).

To date, two curated and validated single-strain genome-scale metabolic models (GEMs) have been reported for *K. pneumoniae*. The first was generated for the MGH78578 laboratory strain and published in 2011 (model ID iYL1228) (Liao et al. 2011). It comprised 1228 genes, 1188 enzymes, and 1970 reactions, and was validated by comparison of in silico growth predictions to true phenotypes generated for 171 substrates using a Biolog phenotyping array. The estimated accuracy of iYL1228 was 84% when compared to Biolog growth phenotypes. A second *K. pneumoniae* GEM, for laboratory strain KPPR1, was published in 2017 (model ID iKp1289) (Henry et al. 2017). This model contained 1289 genes and 2145 reactions. The KPPR1 model was found to be 79% accurate when compared to Biolog phenotype data in terms of predicting substrate-growth phenotypes. More recently, Norsigian and colleagues (Norsigian et al. 2019a) reported nonvalidated draft GEMs for 22 antimicrobial-resistant *K. pneumoniae* clinical isolates built from the iYL1228 model through a subtractive approach. Subsequent in silico growth predictions indicated variability between isolates in terms of carbon, nitrogen, and sulfur, but not phosphorus utilization. There was evidence that nitrogen substrate usage could be used to classify strains associated with distinct drug resistance phenotypes. However, none of these models were experimentally validated.

Here, we present an updated version of the MGH78578 GEM in addition to novel GEMs for 36 KpSC strains, including representatives of all seven taxa in the species complex. We curate and validate the models using a combination of Biolog growth assays and additional targeted growth phenotype data, resulting in a median accuracy of 96%. We define the core reactomes of *K. pneumoniae* and the broader species complex, and we identify species-specific metabolic capabilities. We then explore these models to identify strain-specific gene essentiality and metabolic pathway redundancy across growth on 145 core carbon substrates.

## Results

### Completed KpSC genomes

We collated 37 previously described isolates from the KpSC complex, including at least one representative per taxon (Blin et al. 2017; Rodrigues et al. 2019). The collection spanned a variety of sequence types (STs) within species with more than one strain and represented a wide range of isolation sources (including human-host-associated, water, and the environment). The strains were geographically and temporally diverse, sampled from five continents, and with isolation dates spanning from 1935 to 2010 (Supplemental Table 1).

Eight strains had previously published complete genome sequences available, and we generated complete genome sequences for the remaining 29 strains using a combination of short- and long-read sequencing (Methods). The median genome size was 5.5 Mbp (range 5.1–6.0 Mbp) with a median of 5145 genes (range 4798–5704 genes). The majority of strains carried at least one plasmid (n = 29, 78%), with seven strains carrying five or more plasmids.

### Model generation, curation, and validation

Using these completed genomes we created strain-specific GEMs, initially using the curated MGH78578 GEM (iYL1228) as a reference to identify conserved genes and reactions, followed by manual curation (Methods). The latter was enabled by the availability of matched phenotype data (Blin et al. 2017) indicating the ability of each strain to grow in minimal media supplemented with each of 94 distinct sole carbon substrates for which we were able to predict growth in silico using the GEMs (Supplemental Table 2). Our phenotypic data included 12 carbon substrates for which growth was shown for at least one strain and for which the corresponding metabolite transport and/or processing reactions were not present in the original iYL1228 model. Literature searches were undertaken to identify the putatively responsible candidate genes and reactions for GEM inclusion. For example, all strains were able to use palatinose as a carbon substrate; the reaction required to catabolize this compound was added based on the presence of core genes with ≥99% nucleotide homology with *aglAB* (that encode AglAB), which has been shown to catabolize palatinose in *K. pneumoniae* (Supplemental Table 3; Thompson et al. 2001). When the model-based predictions and our phenotypic growth data disagreed, we attempted to correct the models by identifying alternative pathways from the literature or homologous genes in other *Klebsiella* or Enterobacteriaceae species with sufficient evidence to allow inclusion in our models (Methods; Supplemental Table 3). Overall, we added 49 genes and 56 reactions across all models.

The final curated, validated models were highly accurate for the prediction of growth phenotypes measured through Biolog (median accuracy 95.7%, range 88.3%–96.8%) (Supplemental Table 1). The majority (87%) of the discrepancies were false positives, that is, the model predicted growth on a carbon substrate, but we did not observe any phenotypic growth. False positives usually occur because of gene regulation, in which strains carry the genes encoding the enzymes required to import and metabolize a substrate; however, these genes are not expressed during the phenotypic growth experiments. False positives can also be related to technical issues with measuring metabolic phenotypes, for example, the limit of detection, sensitivity of growth detection, and use of correct standards for measurements (Ibarra et al. 2002). Every model had at least one false positive (median 4, range 1–11) (Supplemental Table 1) across 31 different carbon substrates. The most common false positive calls were predicted growth in 2-oxoglutarate (n = 35 strains), ethanolamine (n = 29), L-ascorbate (n = 28), and 3-hydroxycinnamic acid (n = 20); false positive calls for the remaining 27 carbon substrates were associated with six or fewer strains each (Supplemental Table 4).

Five carbon substrates had at least one strain with a false negative call, in which the model did not predict growth, but we observed a growth phenotype: L-tartaric acid (n = 12 strains), L-lyxose (n = 5), L-sorbose (n = 2), propionic acid (n = 2), and L-galactonic acid-gamma-lactone (n = 1) (Supplemental Table 4). In such cases it is assumed that the models are missing information required to optimize for growth on these substrates (Orth and Palsson 2012). Despite thorough literature and database searches, we were unable to identify alternate biological pathways that could plausibly fill these gaps in the models. This was particularly notable among the five *K. quasipneumoniae* subsp. *quasipneumoniae* strains, which all had false negative predictions for L-lyxose utilization. These genomes were each missing *sgaU* (KPN_04590), which was present in all other KpSC genomes and encodes an enzyme that converts L-ribulose-5-phosphate to L-xylulose-5-phosphate. We were unable to detect any other proteins belonging to this enzyme class or carrying similar domains. As the phenotypic results indicated that all *K. quasipneumoniae* subsp. *quasipneumoniae* can use L-lyxose, we hypothesize that they must contain unknown functional ortholog/s to *sgaU*, which can perform isomerase activity on L-ribulose-5-phosphate.

We performed an independent validation of the models by comparing growth phenotypes from the VITEK GN card with simulated phenotypes (n = 13 substrates; Methods). The models were highly accurate in this setting (median accuracy 100%, range 92.3%–100%) (Supplemental Table 5). All discrepancies were false positives (n = 4): two for growth in succinate, one in tagatose, and one in 5-keto-D-gluconate (Supplemental Table 5).

### Novel GEMs reveal species- and strain-specific metabolic diversity

**Figure 1.**
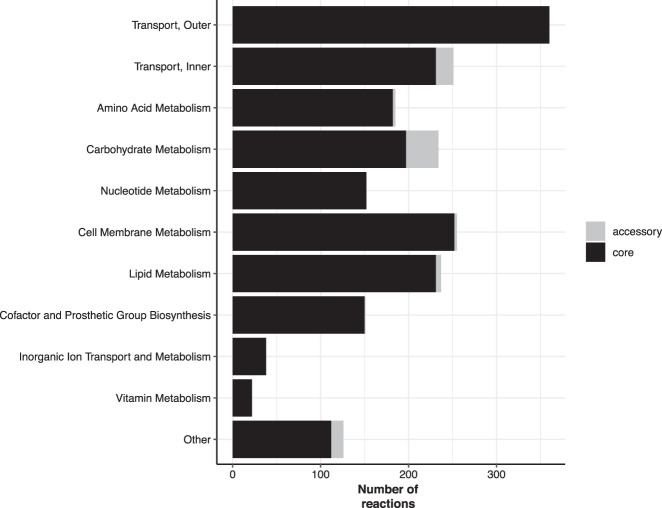
Number of model reactions by category. Bars are colored to indicate core reactions (black, conserved in all strains) and accessory reactions (gray, variably present).

Our strain collection provided us with a novel opportunity to compare predicted metabolic functionality between all seven taxa within the KpSC. Overall there were median 1219 genes and 2294 reactions in each curated strain-specific GEM (ranges 1190–1243 and 2283–2305, respectively), representing median 23.6% of all coding sequences in each genome (Supplemental Table 1). Each species had approximately 1200 core model genes and about 2200 core reactions (Table 1), with a slight decreasing trend with increasing sample size. Conversely, the total number of distinct reactions detected among the best represented species, *K. pneumoniae* (2312, n = 20 genomes), was higher than those detected among each of the species represented by fewer genomes (2299 in *K. quasipneumoniae* subsp. *quasipneumoniae*; 2307 in both *K. quasipneumoniae* subsp. *similipneumoniae*, and *K. variicola* subsp. *variicola*). In terms of the reactions themselves, the vast majority were core across all species (Fig. 1); however, there was variability in reactions associated with carbohydrate metabolism, for which 16% (n = 37/234) were not conserved across all models (Fig. 1). Among these variable reactions we identified three involved in the N-acetylneuraminate pathway (ACNAMt2pp, ACNML, and AMANK) that were species-specific and were found to be core in all five *K. quasipneumoniae* subsp. *similipneumoniae* in our study, but absent from all other genomes. A BLASTN screen of all 307 *K. quasipneumoniae* subsp. *similipneumoniae* genomes from Lam et al. (2021) revealed that these three genes were present in all 307 genomes, indicating that this pathway is likely to be core across all members of the species.

**Table 1.**
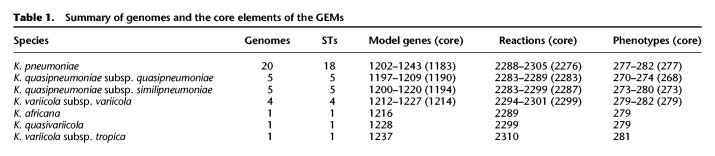
Summary of genomes and the core elements of the GEMs

**Figure 2.**
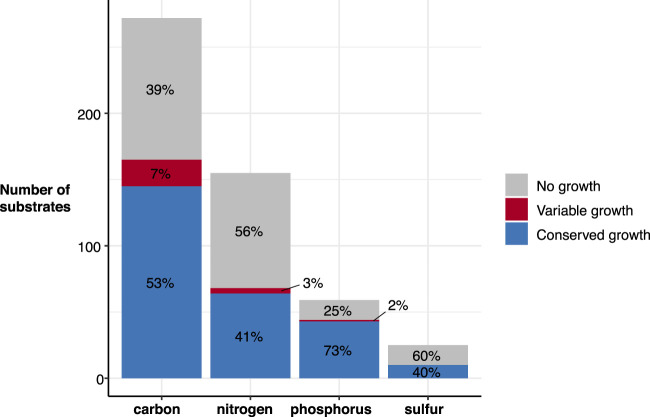
Predicted substrate utilization by type. Bar height indicates number of substrates for each type, with segments colored to indicate those associated with no growth for any strain (gray), variable growth (red), and conserved growth (blue). Percentages are indicated within each segment.

We simulated growth on 511 substrates as the sole sources of either carbon (n = 272), nitrogen (n = 155), phosphorus (n = 59), or sulfur (n = 25) (Methods; Supplemental Table 2). A total of 224 (44%) were unable to support growth for any strain (carbon = 107, nitrogen = 87, phosphorus = 15, sulfur = 15). Overall, the number of core growth-supporting phenotypes was very similar across taxa, with a median of 279 (range 268–281) (Table 1). Of the 287 that were predicted to support growth for at least one strain, 262 were conserved across all 37 strains (carbon = 145, nitrogen = 64, phosphorus = 43, sulfur = 10), with only 25 (5%) substrates variable between strains. Substrates that could be used as a carbon source had the most variation, with 7% of carbon substrates displaying variable predicted growth phenotypes by strain (Fig. 2). This was in stark contrast to substrates used as a source of sulfur, in which no variation was observed (Fig. 2).

**Figure 3.**
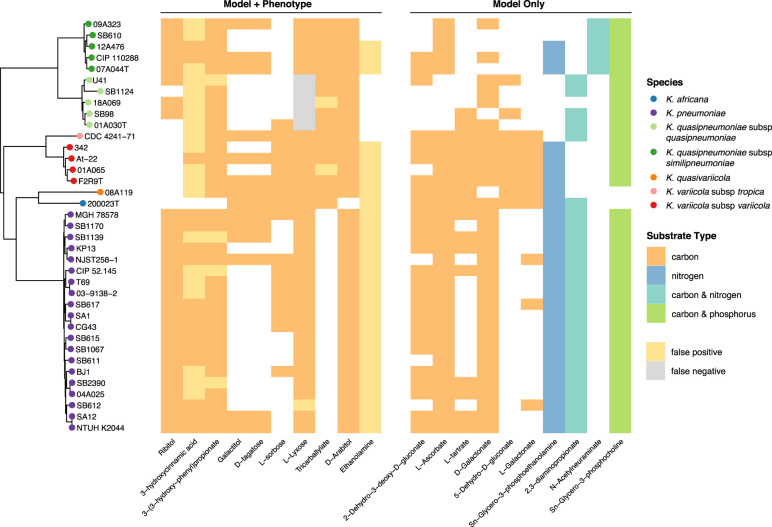
Variable growth phenotypes across all seven taxa in KpSC. (*Left*) Core gene phylogeny for all 37 strains, with tips colored by species per legend. (*Middle*) Heatmap of variable substrates for which both phenotypic growth results and model predicted results were available. White indicates no growth, and color indicates growth. False positive calls are shown in yellow, and false negative calls are in gray (per legend). (*Right*) Heatmap of variable substrates for which only model predictions were available. White indicates no growth, color indicates growth, with substrate type indicated per legend.

Among the 20 variable carbon substrates, there was some species-specific variation. Six of these reflect core growth capabilities in all but one of the seven species (3-hydroxycinnamic acid, 3-(3-hydroxy-phenyl)propionate, D-arabitol, L-ascorbate, L-lyxose, tricarballylate) (Fig. 3). In the case of tricarballylate, we identified a new pathway that was absent from the original *K. pneumoniae* MGH78578 model: all KpSC species except for *K. pneumoniae* carried the *tcuABC* operon, which encodes the enzymes responsible for oxidizing tricarballylate to *cis*-aconitate (Lewis et al. 2009) through the TCBO reaction (Fig. 3). In contrast, all KpSC were able to use L-ascorbate with the exception of *K. quasipneumoniae* subsp. *quasipneumoniae*, in which all five genomes were lacking the *ulaABC* operon encoding the transport reaction ASCBptspp (Fig. 3). This reaction converts L-ascorbate into L-ascorbate-6-phosphate as it is transported into the cytosol (Zhang et al. 2003). We screened all 149 *K. quasipneumoniae* subsp. *quasipneumoniae* genomes from Lam et al. (2021) for *ulaABC* with BLASTN and found that this operon was missing from all members of the species, suggesting that this is a conserved deletion in *K. quasipneumoniae* subsp. *quasipneumoniae*.

The remaining 14 variable carbon substrates were specific to five or fewer strains. For example, sn-glycero-3-phosphocholine could be used by all strains as a carbon and phosphorus substrate, except for the single *K. africana* and *K. quasivariicola* representatives, which share a common ancestor in the core gene phylogenetic tree (Fig. 3). Both these genomes lacked *glpQ*, encoding the enzyme required to convert sn-glycero-3-phosphocholine into sn-glycero-3-phosphate and ethanolamine (Brzoska and Boos 1988). We confirmed that *glpQ* was absent in all 13 *K. quasivariicola* genomes listed in Lam et al. (2021) by screening for the gene using BLASTN. To check the result if the *glpQ* deletion is present in other *K. africana* (because we have only a single genome), we screened six *K. africana* genomes (all ST4838) for *glpQ* from Vezina et al. (2021) and found that this gene was present in all strains. There was only a single carbon substrate, N-acetylneuraminate, which supported growth for all *K. quasipneumoniae* subsp. *similipneumoniae*, because of the presence of the *nan* operon (Vimr and Troy 1985), encoding the proteins required to catalyze the ACNAMt2pp, ACNML, and AMANK reactions, which were absent in all the other species (Fig. 3).

### Single gene knockout simulations reveal variable gene essentiality

Strain-specific GEMs provide an unparalleled opportunity to simulate the impact of single gene knockout mutations for diverse strains. Because carbon substrates were associated with the greatest amount of variation, we focused on the impact of single gene knockouts in this group. For each strain we simulated the impact of deletion of each unique gene in its GEM on growth in each of the core carbon substrates (those predicted to support growth of all strains, n = 145), resulting in 6,544,865 unique simulations (Supplemental Table 6). Among these simulations, 639,365 (9.8%) were predicted to result in a loss-of-growth phenotype.

To compare the diversity of knockout phenotypes between strains, we focused on simulations representing core gene-substrate combinations (n = 164,285 gene-substrate combinations; 1133 genes that were present in all GEMs × 145 substrates) and excluded those representing noncore gene-substrate combinations (n = 19,140 combinations), because the former can be directly compared for all strains, whereas the latter cannot (by definition not all strains harbor all of the genes). A total of 146,385 core gene-substrate combinations (89.1%) resulted in no loss-of-growth phenotype in any strain, whereas 7170 (10.5%) combinations resulted in a loss-of-growth phenotype in all strains. At the gene level, 807 genes (71.2%) were not predicted to be essential for growth for any substrate in any strain, and just 57 genes (5.0%) were predicted to be essential for all substrates in all strains. The latter were associated with 194 distinct reactions (1–32 reactions each, median = 1, Supplemental Table 7), encompassing eight subsystem categories: cell membrane metabolism (n = 76 reactions), lipid metabolism (n = 42), amino acid metabolism (n = 33), transport, inner- (n = 29) or outer-transport (n = 6), nucleotide metabolism (n = 5), carbohydrate metabolism (n = 2), and cofactor and prosthetic group biosynthesis (n = 1).

**Figure 4.**
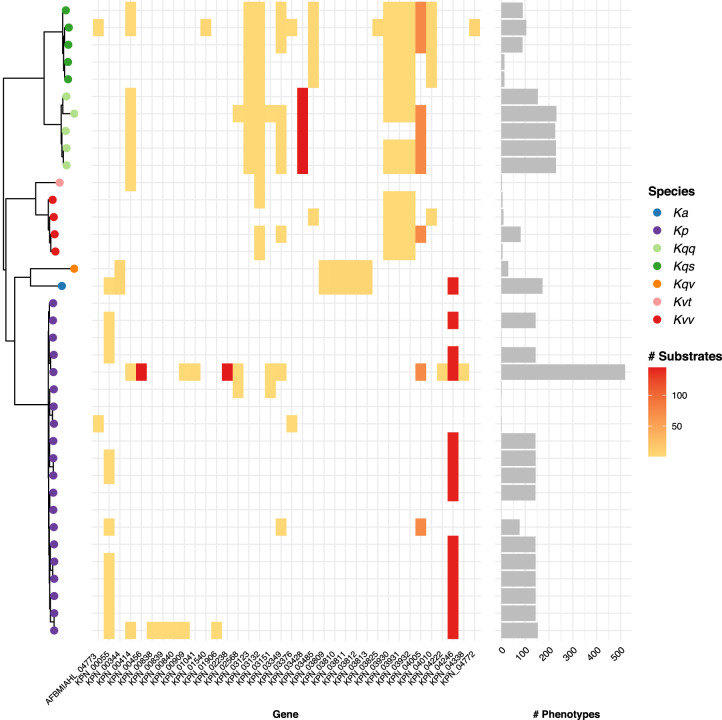
Variable loss-of-growth phenotypes. (*Left*) Core gene phylogeny per Figure 3, with tips colored by species as indicated in legend: (Ka) *K. africana*; (Kp) *K. pneumoniae*; (Kqq) *K. quasipneumoniae* subsp. *quasipneumoniae*; (Kqs) *K. quasipneumoniae* subsp. *similipneumoniae*; (Kqv) *K. quasivariicola*; (Kvt) *K. variicola* subsp. *tropica*; (Kvv) *K. variicola* subsp. *variicola*. (*Middle*) Heatmap showing core genes for which variable loss-of-growth phenotypes were predicted (columns). Shading indicates the number of substrates where loss of growth was predicted for each strain (rows) per the scale legend. (*Right*) Bars show the total number of loss-of-growth phenotypes predicted for each strain.

Gene essentiality varied by strain, with reasonable consistency within species. The number of core gene-substrate combinations predicted to result in a loss-of-growth phenotype ranged from 0 to 519 (median = 143) (Fig. 4), and the number of core genes resulting in a phenotype on at least one growth substrate ranged from 0 to 15 (median = 3). The vast majority of these genes (31 of 36 unique genes, 86.1%) were associated with loss-of-growth phenotypes for six or fewer substrates, with minimal variation in the total number of substrates among those strains that were impacted. In contrast, a small number of genes were associated with loss of growth for all or almost all substrates for some strains (four genes, 11.1%, each affecting 143 or more substrates per strain) (Fig. 4).

We further investigated the core gene deletions predicted to result in loss-of-growth phenotypes for 143 or more substrates in only a subset of strains, beginning with an apparent *K. quasipneumoniae* subsp*. quasipneumoniae* species-specific phenotype. The associated gene, KPN_03428, encodes the enzyme for catalysis of two reactions in the models: CYSDS (cysteine desulfhydrase) and CYSTL (cystathionine b-lyase), the latter of which may also be encoded by KPN_01511 (*malY*). *malY* was present in all other models but absent from all *K. quasipneumoniae* subsp*. quasipneumoniae* (closest bidirectional BLASTP hit had 30.07% identity, well below the threshold required for inclusion as a homolog and considerably lower than the expected divergence between KpSC *s*pecies; 3%–4% nucleotide divergence) (Holt et al. 2015), and no alternate genes encoding putative cystathionine b-lyases could be identified by search of the KEGG database, indicating a lack of genetic redundancy for these reactions. Direct comparison of the *K. quasipneumoniae* subsp. *quasipneumoniae* 01A030T chromosome to *K. pneumoniae* MGH78578 revealed that the former harbored a ∼5 kbp deletion relative to the latter, spanning the *zntB*, *malY*, and *malX* genes as well as part of *malI*. The lack of *malY* (KPN_ 01511) in combination with the KPN_03428 deletion resulted in predicted loss of ability to produce three key metabolites (L-homocysteine, ammonium, and pyruvate) and ultimately the predicted loss of biomass production. This deletion was replicated in all five *K. quasipneumoniae* subsp. *quasipneumoniae* strains. Inspection of an additional 149 publicly available *K. quasipneumoniae* subsp. *quasipneumoniae* genome assemblies (Methods) found this region to be present in only 37 genomes (24%), suggesting that the most recent common ancestor of this species is lacking this region, with occasional reacquisition in some lineages.

Unlike the KPN_03428 deletion, deletion of KPN_04246 resulted in predicted loss-of-growth phenotypes for all 145 substrates for the single *K. africana* strain plus 13 of 20 *K. pneumoniae* strains (comprising multiple distantly related lineages including representatives of the well-known globally distributed ST14, ST23, ST86, and ST258). KPN_04246 encodes a protein that catalyzes two reactions—ACODA, acetylornithine deacetylase, and NACODA, N-acetylornithine deacetylase—both of which may also be encoded by the product of KPN_01464 (homologs of this gene were identified in only those genomes that were not associated with loss-of-growth phenotype). Comparison of the *K. pneumoniae* strain CG43 (ST86) chromosome lacking KPN_01464 to *K. pneumoniae* MGH78578 harboring KPN_01464 showed that CG43 contained a ∼10 kbp deletion resulting in the loss of KPN_01464. This deletion was replicated in the *K. africana* 200023T genome and the remaining 12 *K. pneumoniae* genomes that lacked KPN_01464 (≤33.24% identity for the best bidirectional BLASTP hit, no alternate genes encoding putative acetylornithine deacetylases/N-acetylornithine deacetylases were identified in KEGG).

Finally, we investigated the two gene deletions (KPN_02238 and KPN_00456) resulting in predicted loss of growth on all substrates in only *K. pneumoniae* NJST258-1. KPN_02238 encodes the protein responsible for catalyzing phosphoribosylpyrophosphate synthetase (PRPPS), for which no redundant genes were included in any of our KpSC models. This reaction converts alpha-D-ribose 5-phosphate to 5-phospho-alpha-D-ribose 1-diphosphate, a key substrate used as input for 14 downstream reactions. Although the *K. pneumoniae* MGH78578 reference model contains a redundant pathway to support this conversion, one of the required reactions (R15BPK, catalyzed by a ribose-1,5-bisphosphokinase) was missing from the NJST258-1 model because the associated genome lacked a homolog of KPN_04492 (best bidirection BLASTP hit 26.19% identity), whereas all other genomes contained a homolog of this gene. Further investigation showed that the NJST258-1 chromosome was missing a ∼17 kbp region compared to MGH78578. In the NJST258-1 chromosome, this region, which included KPN_04492, was replaced by the insertion sequence IS*1294* (99% nucleotide identity). We were not able to identify a similar deficiency to explain the strain-specific loss-of-growth phenotype associated with KPN_00456, which encodes a protein implicated in 14 distinct reactions.

## Discussion

Here, we present an updated GEM for *K. pneumoniae* MGH78578 plus novel GEMs for 36 members of the KpSC, capturing all seven taxa and representing the first reported GEMs for the *K. variicola* (subsp. *variicola* and *tropica*), *K. quasipneumoniae* (subsp. *quasipneumoniae* and *similipneumoniae*), *K. quasivariicola*, and *K. africana* species. All models were validated and curated by comparison of predicted and true growth phenotypes, and they had a median accuracy of 95.7% (range 88.3%–96.8%), higher than estimated for the previously published *K. pneumoniae* MGH78578 (84%) and KPPR1 (79%) models.

Our in silico growth phenotype predictions for a diverse set of substrates highlighted variability among strains within the *K. pneumoniae* species, as has been indicated by previous smaller scale GEM comparisons and phenotypic comparisons (Brisse et al. 2009; Blin et al. 2017; Henry et al. 2017; Norsigian et al. 2019a). Similar variability was also indicated within and between the other species in the KpSC (Fig. 3). Carbon substrates were associated with the greatest diversity; a total of 145 substrates (53%) predicted to support growth of all 37 strains and 20 (7%) predicted to support growth of 1–36 strains each (Fig. 2). These predictions were consistent with the observed reaction variability, in which the highest proportion of accessory reactions was identified among those associated with carbohydrate metabolism (16%) (Fig. 1). This is consistent with a previous pan-genome analysis of 328 *K. pneumoniae* that indicated that ∼50% of the total gene pool predicted to encode proteins with metabolic functions were specifically associated with carbohydrate metabolism (Holt et al. 2015). This trend is also consistent with previous studies of the closely related species, *Escherichia coli*, which showed carbohydrate metabolism as the most diverse category for this organism (Monk et al. 2013; Fang et al. 2018).

The extent of diversity reported for *E. coli* and *Salmonella* spp. (Seif et al. 2018) was much higher than reported here for KpSC. We propose two likely explanations for these differences. First, the current analysis for KpSC comprises just 37 strains, compared to 55 and 110 strains included in the *E. coli* studies (Monk et al. 2013; Fang et al. 2018) and 410 in the *Salmonella* study (Seif et al. 2018). With greater sample size we expect to capture greater gene content diversity (Tettelin et al. 2008), including genes associated with metabolic functions that drive metabolic diversity (as was shown to be the case for *Salmonella* spp.) (Seif et al. 2018). Second, our draft KpSC strain-specific models were generated using the reference-based protocol (Norsigian et al. 2019b), in which homology search is used to identify genes in the reference model that are absent from the strain of interest and are therefore removed from the strain-specific model. We added novel genes/reactions to the models based on comparison of predicted versus observed growth phenotypes and manual sequence/literature search, but we did not conduct an automated screen to identify additional genes that are present in the novel strain collection. The latter approach is expected to reveal further diversity, but it requires significant manual curation and validation to ensure the high-quality status of the models is maintained; therefore, it should be addressed in future studies.

In addition to growth capabilities, our analyses revealed considerable variation in terms of predicted gene essentiality as has been implicated for other bacterial species (Breton et al. 2015; Poulsen et al. 2019; Tong et al. 2020; Rousset et al. 2021). Specifically, our data indicate that (1) deletion of a single core gene in a given strain may result in loss of growth on all, none, or only a subset of growth substrates; and (2) the impact of such deletions may vary between strains (Fig. 4). Among genes for which deletion was predicted to have variable impact, most were associated with the loss of growth for only a small number of substrates in the impacted strains. However, four genes were associated with predicted loss of growth on 143 or more of 145 substrates for between one and 14 strains each. In two cases (genes KPN_03428 and KPN_04246), the impacted strains were missing redundant genes that were present in the MGH78578 reference model—that is, those encoding proteins with the same functional annotation as the deleted gene. Comparisons of the chromosomes of these strains suggested that the genes were lost through large-scale chromosomal deletions (5–10 kbp). One of these deletions was uniquely conserved among strains belonging to *K. quasipneumoniae* subsp. *quasipneumoniae*, suggesting that it may have occurred in the most recent common ancestor of this subspecies and has been inherited through vertical descent, with evidence from additional public genome data pointing toward recent reacquisition of this region in some lineages. The other chromosomal deletion was found among a distantly related subset of *K. pneumoniae* as well as the single *K. africana* isolate; therefore, its distribution cannot be explained by simple vertical ancestry. Rather, we speculate that this deletion has been disseminated horizontally by chromosomal recombination, as is known to occur frequently among *K. pneumoniae* (Bowers et al. 2015; Wyres et al. 2019) and has been reported between KpSC species (Holt et al. 2015).

Deletion of two genes (KPN_02238 and KPN_00456) resulted in the loss of growth on all substrates for only a single strain (*K. pneumoniae* NJST258-1). This strain is of particular interest because it was associated with the highest number of deletion phenotypes (Fig. 4), and it belongs to ST258, a globally distributed cause of carbapenem-resistant *K. pneumoniae* infections (Bowers et al. 2015; Wyres et al. 2020). We were unable to identify the cause of this rare knockout phenotype (lacking adenylate kinase, encoded by KPN_00456), which converts D-ribose 1,5-bisphosphate to 5-phospho-alpha-D-ribose 1-diphosphate at the cost of 1 ATP. Comparison of the metabolic networks of NJST258-1 and MGH78578 indicated that NJST258-1 was lacking an additional reaction pathway (phosphoribosylpyrophosphate synthetase) present in MGH78578, allowing an alternative means of 5-phospho-alpha-D-ribose 1-diphosphate production in the absence of ribose-1,5-bisphosphokinase. Further investigation showed that the NJST258-1 chromosome was missing a ∼17 kbp region containing one of the genes required to express this redundant pathway, which had been replaced by an insertion sequence (IS). ISs are frequently identified among *Klebsiella* and other Enterobacteriaceae where they are particularly associated with large plasmids and the dissemination of antimicrobial resistance (Adams et al. 2016; Che et al. 2021). The carbapenem-resistant *K. pneumoniae* lineage, ST258, has been associated with particularly high IS burden (Adams et al. 2016), and we hypothesize that such insertions contribute to the increased number of gene deletion phenotypes predicted for NJST258-1 compared to other *K. pneumoniae* strains. We screened an additional 1021 nonredundant ST258 genomes from Lam et al. (2021) for the presence of KPN_02388 (the gene which encodes for phosphoribosylpyrophosphate synthetase) and found that this gene was present in all 1021 ST258 genomes, suggesting that the deletion of this pathway is unique to NJST258-1. This highlights the importance of assessing multiple strains when attempting to draw conclusions regarding observed phenotypes.

These findings indicate that KpSC can differ substantially in terms of metabolic redundancy. We cannot exclude the possibility that the predicted knockout phenotypes might be rescued by products of nonorthologous genes that are not currently captured in our models, but at least for the aforementioned examples, a search of the KEGG database did not indicate any additional known redundant metabolic pathways. Additionally, our findings are consistent with a recent experimental exploration of gene essentiality in *E. coli* (Rousset et al. 2021), which showed that 7%–9% of roughly 3400 conserved genes were variably essential among 18 *E. coli* strains grown in three different conditions. Genomic comparisons of these *E. coli* implicated a key role for horizontal gene transfer in driving strain-specific essentiality patterns and redundancies through the mobilization of homologous or analogous genes and/or those driving epistatic interactions (Rousset et al. 2021).

Taken together our findings highlight the importance of strain-specific genomic variation in determining strain-specific metabolic traits and redundancy. More broadly, these analyses show the value of an organism investing in redundant systems, either through (1) encoding multiple genes capable of performing the same reaction, or through (2) encoding multiple, alternative pathways for producing key metabolites from different substrates. Given what is known about the extent of genomic diversity among *K. pneumoniae* and the broader KpSC (Holt et al. 2015; Wyres et al. 2019; Thorpe et al. 2021), it is clear that studies seeking to understand the metabolism of these species—for example, for novel drug design, or to identify novel virulence and drug resistance determinants—should include a diverse set of strains. In this regard, we anticipate that the GEMs, growth predictions, and single gene deletion predictions presented here will provide a valuable resource to the *Klebsiella* research community that can be used to understand the fundamental biology of these organisms and to derive clinically relevant insights—for example, to understand how substrate usage patterns influence pathogenicity and virulence, or to identify universal or clone-specific metabolic choke points wherein the associated essential genes/proteins could be targeted by novel therapeutics. As exemplified for the *E. coli* K-12 reference strain, such resources can be continually improved and expanded to maximize their utility and facilitate biological discovery for years to come (Schilling et al. 1999; Monk et al. 2017).

## Methods

### Genome collection

The 37 strains used in this study were sourced from two previous studies (Blin et al. 2017; Rodrigues et al. 2019). Eight strains had completed genome sequences already publicly available, generated using various sequencing and assembly methods (for details, see Supplemental Table 1). For the remaining 29 strains, short- and long-read sequencing was conducted as follows. Genomic DNA was extracted from overnight cultures, using GenFind v3 reagents (Beckman Coulter). The same DNA extraction was used for both Illumina and MinION libraries. Illumina sequencing libraries were made with Illumina DNA Prep reagents (catalog no. 20018705) and the Illumina Nextera DNA UD Indexes (catalog no. 20027217) per manufacturer's instructions with one major deviation from described protocol: Reactions were scaled down to 25% of recommended usage. Illumina libraries were sequenced on the NovaSeq platform using the 6000 SP Reagent Kit (300 cycles; catalog no. 20027465), generating 250-bp paired-end reads. A total of 21 strains were sequenced across multiple long-read sequencing libraries, prepared using the ligation library kit (LSK-109, Oxford Nanopore Technologies [ONT]) with native barcoding expansion pack (EXP-NBD104 and NBD114, ONT). The libraries were run on a R9.4.1 MinION flow cell and base called with Guppy v3.3.3 using the dna_r9.4.1_450 bps_hac (high-accuracy) base calling model. The remaining seven strains had their DNA extracted using Qiagen Genomic DNA kits (Qiagen Genomic-tip 100/G) and sequenced using Pacific Biosciences (PacBio) RS II.

The Illumina and MinION read data were combined to generate completed genomes for n = 28/29 strains with Unicycler v0.4.8 (Wick et al. 2017) using default parameters. SB610 could not be assembled into a completed genome using this approach, so we used Trycycler v0.3.3 (Wick et al. 2021) to combine 12 independent long-read only assemblies into a single consensus assembly. The 12 assemblies were generated from 12 independent subsets of the long reads (randomly selected) at 50× depth, which were assembled with one of three assemblers (n = 4 assemblies each): Flye v2.7 (Kolmogorov et al. 2019), Raven v1.1.10 (Vaser and Šikić 2021), and Miniasm v0.3 (Li 2016). The final consensus assembly was then polished with the long reads using Medaka v1.1.3 (https://github.com/nanoporetech/medaka), followed by three rounds of polishing using the Illumina reads with Pilon v1.23 (Walker et al. 2014). The PacBio reads were assembled with HGAP, and overlaps between contigs extremities were manually circularized. All 37 completed genomes were annotated with Prokka v1.13.3 (Seemann 2014), using a trained annotation model (created using 10 genomes with Prodigal v2.6.3) (Hyatt et al. 2010). All genomes were analyzed with Kleborate v2.0.3 (Lam et al. 2021) to obtain ST and other genomic information (Supplemental Table 1).

### Phenotypic testing

We used the Biolog growth phenotypes for 190 carbon substrates generated previously (Blin et al. 2017; Rodrigues et al. 2019). As determined in Blin et al. (2017), a maximum value in the respiration curve of 150 or greater was used to indicate growth, whereas a value of less than 150 indicated no growth.

We performed additional phenotypic tests on six carbon substrates; two which were not available on Biolog, 3-(3-hydroxy-phenyl)propionate (Sigma-Aldrich PH011597) and 3-hydroxycinnamic acid (CAS 14755-02-3); and four Biolog substrates for which we required further evidence, gamma-amino butyric acid (CAS 56-12-2), L-sorbose (CAS 87-79-6), D-galactarate (CAS 526-99-8), and tricarballylate (CAS 99-14-9). Overnight cultures of all 37 isolates were grown in M9 minimal media (2× M9, Minimal Salts [Sigma-Aldrich], 2 mM MgSO_4_, and 0.1 mM CaCl_2_) plus 20 mM D-glucose, at 37°C, shaking at 200 RPM. Each carbon source substrate solution was prepared to a final concentration of 20 mM in M9 minimal media, pH 7.0. Then, 200 µL of each substrate solution was added to separate 96-well cell culture plates (Corning) and 5 µL of overnight cultures added to the wells, diluted to McFarland standard of 0.4–0.55. Negative controls were included on every independent plate and included (1) no substrate solution controls (20 mM M9 minimal media) and (2) no isolate controls but 20 mM substrate solution. For positive controls, each isolate was also grown independently in M9 minimal media containing 20 mM D-glucose. Every growth condition was performed in technical triplicate. Plates were then sealed with AeraSeal film (Sigma-Aldrich) and then grown aerobically for 18 h at 37°C, shaking at 200 RPM. Plates were then read using the FLUOstar Omega plate reader (BMG Labtech) using Read Control version 5.50 R4, firmware version 1.50, using 595 nm absorbance after 30 sec of shaking at 200 RPM. No isolate controls were used as blanks to generate the optical density (OD) value for each technical replicate. We then averaged all no isolate OD values to obtain a mean M9 media OD value. To determine growth/no growth using the OD method, we calculated the mean OD for growth on a particular substrate for each strain at 24 h and subtracted from this the OD value of M9 media alone. Subsequently, for each carbon substrate, we divided the mean OD value for a strain by the mean OD for that strain in M9 media alone to get an OD fold change. OD fold changes ≥2 were considered sufficient evidence of growth (Supplemental Fig. 1).

We performed additional growth tests for independent validation of the models using VITEK 2 GN ID cards (bioMérieux). All 37 strains were assayed on the card to evaluate growth on 13 carbon sources (VITEK codes for those 13 sources can be found in Supplemental Table 5). Cards were read on the VITEK 2 Compact (bioMérieux) per the manufacturer's instructions using VITEK 2 software version 8.0.

### Creating and curating strain-specific GEMs

Using the method outlined by Norsigian et al. (2019b), we extracted and translated all CDS from each genome and used bidirectional BLASTP hits (BBH) to determine orthologous genes compared to the reference *K. pneumoniae* MGH78578 GEM (iYL1228) (Liao et al. 2011). Genes with at least 75% amino acid identity were considered orthologous. Genes and their reactions that did not meet this threshold were removed from their respective models.

During GEM creation, we discovered that the original biomass function (BIOMASS_) in iYL1228 required the production of both rhamnose, which is a component of the capsule in *K. pneumoniae* MGH 78578, as well as UDP-galacturonate and UDP-galactose, which are components of the variable O antigen. Because both the capsule and O antigens are known to differ greatly between strains (Follador et al. 2016; Wyres et al. 2016), we created a new biomass function (BIOMASS_Core_Oct2019) that no longer required the associated metabolites dtdprmn_c, udpgalur_c, and udpgal_c.

To validate each GEM against its respective phenotypic growth results, we used flux-based analysis (FBA) implemented in the COBRApy framework (Ebrahim et al. 2013) to simulate growth of each GEM in M9 media with all possible sole carbon, nitrogen, phosphorus, or sulfur substrates. The updated BIOMASS function, BIOMASS_Core_Oct2019, was used as the objective to be optimized. M9 media was defined by setting the lower bound of the cob(I)alamin exchange reaction to −0.01, and the lower bound of the following exchange reactions to −1000: Ca^2+^, Cl^−^, CO_2_, Co^2+^, Cu^2+^, Fe^2+^, Fe^3+^, H^+^, H_2_O, K^+^, Mg^2+^, Mn^2+^, MoO_4_^2−^, Na^+^, Ni^2+^, Zn^2+^. To predict growth on alternate carbon substrates, we set the lower bound of glucose to zero (to prevent the model using this as a carbon source), and then set the lower bound of all potential carbon substrates to −1000 in turn. The carbon substrate was considered growth supporting if the predicted growth rate was ≥0.001. The code used to simulate growth on each substrate can be found in Supplemental Code (simulate_ growth_single.py).

While identifying carbon substrates, the default nitrogen, phosphorus, and sulfur substrates were ammonium (NH_4_), inorganic phosphate (HPO_4_), and inorganic sulfate (SO_4_). Prediction of nitrogen, phosphorus, and sulfur supporting substrates was performed in the same way as carbon but setting glucose as the default carbon substrate.

We matched predictions and phenotypic growth data for all strains for 94 distinct carbon substrates. These data were used to (1) curate and update the models and (2) estimate model accuracy. Where we had evidence of phenotypic growth but a lack of simulated growth, we attempted to identify the missing reactions using gene homology searches and literature searches in related bacteria (for a full list of reactions added and the evidence for each, see Supplemental Table 3). During this process it became apparent that the directionality of the following transport reactions in the original iYL1228 GEM were set to export the compound from the cell rather than allow uptake (TARTRtex, SUCCtex, FORtex, FUMtex, THRtex, ACMANAtex, MALDtex, ABUTtex, AKGtex). Each of these reactions were updated to be reversible (bound range −1000 to 1000), restoring the ability for the model to use the associated compounds.

Strain model accuracy was determined by calculating the percentage of true positive and negative compounds, as well as calculating Matthew's correlation coefficient using the following formula (TP = true positive; TN = true negative; FP = false positive; FN = false negative):

$$\frac{TP\times TN-FP\times FN}{\sqrt{\left(TP+FP\right)\left(TP+FN\right)\left(TN+FP\right)\left(TN+FN\right)}}.$$

We assessed accuracy against the VITEK growth data using the same method as described previously. However, these data were used only to estimate model accuracy and were not used to curate or update the models.

### Gene essentiality for growth on core carbon substrates

To determine which genes were essential for growth in each core carbon substrate (n = 145) for each strain, we used the *single_gene_ deletion* functions in COBRAPy (Ebrahim et al. 2013). For each GEM, on every core carbon substrate we simulated growth in M9 media with that substrate as the sole carbon source using FBA (as described previously), but with one gene knocked out using the *single_gene_deletion* function. Each gene was knocked out in turn, and optimized biomass values ≥0.001 were considered positive for growth. The code used to perform the knockouts and growth simulations on each substrate can be found in Supplemental Code (single_gene_knockouts.py).

Four gene-substrate combinations were selected for further investigation by interrogation of the model gene-protein-reaction rules and search of the KEGG database (Kanehisa et al. 2002) using KofamKOALA (Aramaki et al. 2020) for redundant genes/pathways. When relevant, pairwise chromosomal comparisons were performed using BLASTN (Camacho et al. 2009) and visualized using the Artemis Comparison Tool (Carver et al. 2005). The putative insertion sequence was identified by BLASTN search of the ISFinder database (Siguier et al. 2006).

### Core genome phylogeny

The core genome for the set of 37 genomes was determined using panaroo v1.1.2 (Tonkin-Hill et al. 2020) in strict mode with a gene homology cutoff of 90% identity, which generated a core gene alignment consisting of 3717 genes with 75,899 variable sites. We generated a phylogeny using this core gene alignment with IQ-Tree v2 (Minh et al. 2020), which selected GTR + F+I + G4 as the best-fit substitution model. The resulting phylogeny was visualized using *ggtree* (Yu et al. 2017) in R (R Core Team 2021).

### Gene screening in public genomes

To determine whether specific gene deletions or acquisitions are likely to be conserved in all members of a species or clone, we used the curated set of 13,156 *Klebsiella* genome assemblies from Lam et al. (2021). We used BLASTN to screen for (1) the *nan* operon in 307 *K. quasipneumoniae* subsp. *similipneumoniae* genomes; (2) the *ulaABC* operon in 149 *K. quasipneumoniae* subsp. *quasipneumoniae* genomes; (3) *glpQ* in 13 *K. quasivariicola* genomes and six *K. africana* genomes (Vezina et al. 2021); and (4) KPN_02388 in 1021 nonredundant ST258 genomes. Hits with ≥90% coverage and ≥90% identity were considered to be present.

## Data access

All completed genomes and raw sequence data generated in this study have been submitted to the NCBI BioProject database (BioProject; https://www.ncbi.nlm.nih.gov/bioproject/) under accession number PRJNA768294. All strain metabolic models generated in this study have been deposited in JSON format, along with the gene annotations used for the models in figshare (https://doi.org/10.26180/16702840). MEMOTE reports for all models can be found in figshare (https://doi.org/10.26180/19180274.v1).

## Supplementary Material

Supplemental Material

## References

[GR276289HAWC1] Adams MD, Bishop B, Wright MS. 2016. Quantitative assessment of insertion sequence impact on bacterial genome architecture. Microb Genom 2: e000062. 10.1099/mgen.0.000062 28348858 PMC5343135

[GR276289HAWC2] Aramaki T, Blanc-Mathieu R, Endo H, Ohkubo K, Kanehisa M, Goto S, Ogata H. 2020. KofamKOALA: KEGG ortholog assignment based on profile HMM and adaptive score threshold. Bioinformatics 36: 2251–2252. 10.1093/bioinformatics/btz859 31742321 PMC7141845

[GR276289HAWC3] Bartell JA, Blazier AS, Yen P, Thøgersen JC, Jelsbak L, Goldberg JB, Papin JA. 2017. Reconstruction of the metabolic network of *Pseudomonas aeruginosa* to interrogate virulence factor synthesis. Nat Commun 8: 14631. 10.1038/ncomms14631 28266498 PMC5344303

[GR276289HAWC4] Blin C, Passet V, Touchon M, Rocha EPC, Brisse S. 2017. Metabolic diversity of the emerging pathogenic lineages of *Klebsiella pneumoniae*. Environ Microbiol 19: 1881–1898. 10.1111/1462-2920.13689 28181409

[GR276289HAWC5] Bosi E, Monk JM, Aziz RK, Fondi M, Nizet V, Palsson BØ. 2016. Comparative genome-scale modelling of *Staphylococcus aureus* strains identifies strain-specific metabolic capabilities linked to pathogenicity. Proc Natl Acad Sci 113: E3801–E3809. 10.1073/pnas.1523199113 27286824 PMC4932939

[GR276289HAWC6] Bowers JR, Kitchel B, Driebe EM, MacCannell DR, Roe C, Lemmer D, de Man T, Rasheed JK, Engelthaler DM, Keim P, 2015. Genomic analysis of the emergence and rapid global dissemination of the clonal group 258 *Klebsiella pneumoniae* pandemic. PLoS One 10: e0133727. 10.1371/journal.pone.0133727 26196384 PMC4510304

[GR276289HAWC7] Breton YL, Belew AT, Valdes KM, Islam E, Curry P, Tettelin H, Shirtliff ME, El-Sayed NM, McIver KS. 2015. Essential genes in the core genome of the human pathogen *Streptococcus pyogenes*. Sci Rep 5: 9838. 10.1038/srep09838 25996237 PMC4440532

[GR276289HAWC8] Brisse S, Fevre C, Passet V, Issenhuth-Jeanjean S, Tournebize R, Diancourt L, Grimont P. 2009. Virulent clones of *Klebsiella pneumoniae*: identification and evolutionary scenario based on genomic and phenotypic characterization. PLoS One 4: e4982. 10.1371/journal.pone.0004982 19319196 PMC2656620

[GR276289HAWC9] Brzoska P, Boos W. 1988. Characteristics of a *ugp*-encoded and *phoB*-dependent glycerophosphoryl diester phosphodiesterase which is physically dependent on the *ugp* transport system of *Escherichia coli*. J Bacteriol 170: 4125–4135. 10.1128/jb.170.9.4125-4135.1988 2842304 PMC211418

[GR276289HAWC10] Camacho C, Coulouris G, Avagyan V, Ma N, Papadopoulos J, Bealer K, Madden TL. 2009. BLAST+: architecture and applications. BMC Bioinformatics 10: 421. 10.1186/1471-2105-10-421 20003500 PMC2803857

[GR276289HAWC11] Carver TJ, Rutherford KM, Berriman M, Rajandream MA, Barrell BG, Parkhill J. 2005. ACT: the Artemis comparison tool. Bioinformatics 21: 3422–3423. 10.1093/bioinformatics/bti553 15976072

[GR276289HAWC12] Che Y, Yang Y, Xu X, Břinda K, Polz MF, Hanage WP, Zhang T. 2021. Conjugative plasmids interact with insertion sequences to shape the horizontal transfer of antimicrobial resistance genes. Proc Natl Acad Sci 118: e2008731118. 10.1073/pnas.2008731118 33526659 PMC8017928

[GR276289HAWC13] Ebrahim A, Lerman JA, Palsson BO, Hyduke DR. 2013. COBRApy: COnstraints-Based Reconstruction and Analysis for Python. BMC Syst Biol 7: 74. 10.1186/1752-0509-7-74 23927696 PMC3751080

[GR276289HAWC14] Fang X, Monk JM, Mih N, Du B, Sastry AV, Kavvas E, Seif Y, Smarr L, Palsson BO. 2018. *Escherichia coli* B2 strains prevalent in inflammatory bowel disease patients have distinct metabolic capabilities that enable colonization of intestinal mucosa. BMC Syst Biol 12: 66. 10.1186/s12918-018-0587-5 29890970 PMC5996543

[GR276289HAWC15] Follador R, Heinz E, Wyres KL, Ellington MJ, Kowarik M, Holt KE, Thomson NR. 2016. The diversity of *Klebsiella pneumoniae* surface polysaccharides. Microb Genom 2: e000073. 10.1099/mgen.0.000073 28348868 PMC5320592

[GR276289HAWC16] Gorrie CL, Mirčeta M, Wick RR, Edwards DJ, Strugnell RA, Pratt N, Garlick J, Watson K, Pilcher D, McGloughlin S, 2017. Gastrointestinal carriage is a major reservoir of *Klebsiella pneumoniae* infection in intensive care patients. Clin Inf Dis 65: 208–215. 10.1093/cid/cix270 PMC5850561 28369261

[GR276289HAWC17] Henry CS, Rotman E, Lathem WW, Tyo KEJ, Hauser AR, Mandel MJ. 2017. Generation and validation of the iKp1289 metabolic model for *Klebsiella pneumoniae* KPPR1. J Infect Dis 215: S37–S43. 10.1093/infdis/jiw465 28375518 PMC5790149

[GR276289HAWC18] Holt KE, Wertheim H, Zadoks RN, Baker S, Whitehouse CA, Dance D, Jenney A, Connor TR, Hsu LY, Severin J, 2015. Genomic analysis of diversity, population structure, virulence, and antimicrobial resistance in *Klebsiella pneumoniae*, an urgent threat to public health. Proc Natl Acad Sci 112: E3574–E3581. 10.1073/pnas.1501049112 26100894 PMC4500264

[GR276289HAWC19] Hyatt D, Chen GL, LoCascio PF, Land ML, Larimer FW, Hauser LJ. 2010. Prodigal: prokaryotic gene recognition and translation initiation site identification. BMC Bioinformatics 11: 119. 10.1186/1471-2105-11-119 20211023 PMC2848648

[GR276289HAWC20] Ibarra RU, Edwards JS, Palsson BO. 2002. *Escherichia coli* K-12 undergoes adaptive evolution to achieve in silico predicted optimal growth. Nature 420: 186–189. 10.1038/nature01149 12432395

[GR276289HAWC21] Jung HM, Jung MY, Oh MK. 2015. Metabolic engineering of *Klebsiella pneumoniae* for the production of *cis, cis*-muconic acid. Appl Microbiol Biot 99: 5217–5225. 10.1007/s00253-015-6442-3 25681152

[GR276289HAWC22] Kanehisa M, Goto S, Kawashima S, Nakaya A. 2002. The KEGG databases at genomeNet. Nucleic Acids Res 30: 42–46. 10.1093/nar/30.1.42 11752249 PMC99091

[GR276289HAWC23] Kolmogorov M, Yuan J, Lin Y, Pevzner PA. 2019. Assembly of long, error-prone reads using repeat graphs. Nat Biotechnol 37: 540–546. 10.1038/s41587-019-0072-8 30936562

[GR276289HAWC24] Lam MMC, Wick RR, Watts SC, Cerdeira LT, Wyres KL, Holt KE. 2021. A genomic surveillance framework and genotyping tool for *Klebsiella pneumoniae* and its related species complex. Nat Commun 12: 4188. 10.1038/s41467-021-24448-3 34234121 PMC8263825

[GR276289HAWC25] Lewis JA, Stamper LW, Escalante-Semerena JC. 2009. Regulation of expression of the tricarballylate utilization operon (*tcuABC*) of *Salmonella enterica*. Res Microbiol 160: 179–186. 10.1016/j.resmic.2009.01.001 19284970 PMC2692759

[GR276289HAWC26] Li H. 2016. Minimap and miniasm: fast mapping and de novo assembly for noisy long sequences. Bioinformatics 32: 2103–2110. 10.1093/bioinformatics/btw152 27153593 PMC4937194

[GR276289HAWC27] Li Y, Wang X, Ge X, Tian P. 2016. High production of 3-hydroxypropionic acid in *Klebsiella pneumoniae* by systematic optimization of glycerol metabolism. Sci Rep 6: 26932. 10.1038/srep26932 27230116 PMC4882505

[GR276289HAWC28] Liao YC, Huang TW, Chen FC, Charusanti P, Hong JSJ, Chang HY, Tsai SF, Palsson BO, Hsiung CA. 2011. An experimentally validated genome-scale metabolic reconstruction of *Klebsiella pneumoniae* MGH 78578, *i*YL1228. J Bacteriol 193: 1710–1717. 10.1128/JB.01218-10 21296962 PMC3067640

[GR276289HAWC29] Long SW, Olsen RJ, Eagar TN, Beres SB, Zhao P, Davis JJ, Brettin T, Xia F, Musser JM. 2017. Population genomic analysis of 1,777 extended-spectrum beta-lactamase-producing *Klebsiella pneumoniae* isolates, Houston, Texas: unexpected abundance of clonal group 307. mBio 8: e00489-17. 10.1128/mBio.00489-17 28512093 PMC5433097

[GR276289HAWC30] Minh BQ, Schmidt HA, Chernomor O, Schrempf D, Woodhams MD, von Haeseler A, Lanfear R. 2020. IQ-TREE 2: new models and efficient methods for phylogenetic inference in the genomic era. Mol Biol Evol 37: 1530–1534. 10.1093/molbev/msaa015 32011700 PMC7182206

[GR276289HAWC31] Monk JM, Charusanti P, Aziz RK, Lerman JA, Premyodhin N, Orth JD, Feist AM, Palsson BØ. 2013. Genome-scale metabolic reconstructions of multiple *Escherichia coli* strains highlight strain-specific adaptations to nutritional environments. Proc Natl Acad Sci 110: 20338–20343. 10.1073/pnas.1307797110 24277855 PMC3864276

[GR276289HAWC32] Monk JM, Lloyd CJ, Brunk E, Mih N, Sastry A, King Z, Takeuchi R, Nomura W, Zhang Z, Mori H, 2017. *i*ML1515, a knowledgebase that computes *Escherichia coli* traits. Nat Biotechnol 35: 904–908. 10.1038/nbt.3956 29020004 PMC6521705

[GR276289HAWC33] Navon-Venezia S, Kondratyeva K, Carattoli A. 2017. *Klebsiella pneumoniae*: a major worldwide source and shuttle for antibiotic resistance. FEMS Microbiol Rev 41: 252–275. 10.1093/femsre/fux013 28521338

[GR276289HAWC34] Norsigian CJ, Attia H, Szubin R, Yassin AS, Palsson BØ, Aziz RK, Monk JM. 2019a. Comparative genome-scale metabolic modeling of metallo-β-lactamase–producing multidrug-resistant *Klebsiella pneumoniae* clinical isolates. Front Cell Infect Microbiol 9: 161. 10.3389/fcimb.2019.00161 31179245 PMC6543805

[GR276289HAWC35] Norsigian CJ, Fang X, Seif Y, Monk JM, Palsson BO. 2019b. A workflow for generating multi-strain genome-scale metabolic models of prokaryotes. Nat Protoc 15: 1–14. 10.1038/s41596-019-0254-3 31863076 PMC7017905

[GR276289HAWC36] O'Brien EJ, Monk JM, Palsson BO. 2015. Using genome-scale models to predict biological capabilities. Cell 161: 971–987. 10.1016/j.cell.2015.05.019 26000478 PMC4451052

[GR276289HAWC37] Orth JD, Palsson B. 2012. Gap-filling analysis of the *i*JO1366 *Escherichia coli* metabolic network reconstruction for discovery of metabolic functions. BMC Syst Biol 6: 30. 10.1186/1752-0509-6-30 22548736 PMC3423039

[GR276289HAWC38] Pendleton JN, Gorman SP, Gilmore BF. 2013. Clinical relevance of the ESKAPE pathogens. Expert Rev Anti Infect Ther 11: 297–308. 10.1586/eri.13.12 23458769

[GR276289HAWC39] Poulsen BE, Yang R, Clatworthy AE, White T, Osmulski SJ, Li L, Penaranda C, Lander ES, Shoresh N, Hung DT. 2019. Defining the core essential genome of *Pseudomonas aeruginosa*. Proc Natl Acad Sci 116: 10072–10080. 10.1073/1900570116 31036669 PMC6525520

[GR276289HAWC40] Ramos PIP, Fernández Do Porto D, Lanzarotti E, Sosa EJ, Burguener G, Pardo AM, Klein CC, Sagot MF, de Vasconcelos ATR, Gales AC, 2018. An integrative, multi-omics approach towards the prioritization of *Klebsiella pneumoniae* drug targets. Sci Rep 8: 10755. 10.1038/s41598-018-28916-7 30018343 PMC6050338

[GR276289HAWC041] R Core Team. 2021. R: a language and environment for statistical computing. R Foundation for Statistical Computing, Vienna. https://www.R-project.org/.

[GR276289HAWC41] Rodrigues C, Passet V, Rakotondrasoa A, Diallo TA, Criscuolo A, Brisse S. 2019. Description of *Klebsiella africanensis* sp. nov., *Klebsiella variicola* subsp. *tropicalensis* subsp. nov. and *Klebsiella variicola* subsp. *variicola* subsp. nov. Res Microbiol 170: 165–170. 10.1016/j.resmic.2019.02.003 30817987

[GR276289HAWC42] Rousset F, Cabezas-Caballero J, Piastra-Facon F, Fernández-Rodríguez J, Clermont O, Denamur E, Rocha EPC, Bikard D. 2021. The impact of genetic diversity on gene essentiality within the *Escherichia coli* species. Nat Microbiol 6: 301–312. 10.1038/s41564-020-00839-y 33462433

[GR276289HAWC43] Schilling CH, Edwards JS, Palsson BO. 1999. Toward metabolic phenomics: analysis of genomic data using flux balances. Biotechnol Progr 15: 288–295. 10.1021/bp9900357 10356245

[GR276289HAWC44] Seemann T. 2014. Prokka: rapid prokaryotic genome annotation. Bioinformatics 30: 2068–2069. 10.1093/bioinformatics/btu153 24642063

[GR276289HAWC45] Seif Y, Kavvas E, Lachance JC, Yurkovich JT, Nuccio SP, Fang X, Catoiu E, Raffatellu M, Palsson BO, Monk JM. 2018. Genome-scale metabolic reconstructions of multiple *Salmonella* strains reveal serovar-specific metabolic traits. Nat Commun 9: 3771. 10.1038/s41467-018-06112-5 30218022 PMC6138749

[GR276289HAWC46] Siguier P, Perochon J, Lestrade L, Mahillon J, Chandler M. 2006. ISfinder: the reference centre for bacterial insertion sequences. Nucleic Acids Res 34: D32–D36. 10.1093/nar/gkj014 16381877 PMC1347377

[GR276289HAWC47] Tettelin H, Riley D, Cattuto C, Medini D. 2008. Comparative genomics: the bacterial pan-genome. Curr Opin Microbiol 11: 472–477. 10.1016/j.mib.2008.09.006 19086349

[GR276289HAWC48] Thiele I, Palsson BØ. 2010. A protocol for generating a high-quality genome-scale metabolic reconstruction. Nat Protoc 5: 93–121. 10.1038/nprot.2009.203 20057383 PMC3125167

[GR276289HAWC49] Thompson J, Robrish SA, Immel S, Lichtenthaler FW, Hall BG, Pikis A. 2001. Metabolism of sucrose and its five linkage-isomeric α-d-glucosyl-d-fructoses by *Klebsiella pneumoniae*. J Biol Chem 276: 37415–37425. 10.1074/jbc.M106504200 11473129

[GR276289HAWC50] Thorpe H, Booton R, Kallonen T, Gibbon MJ, Couto N, Passet V, Fernandez JSL, Rodrigues C, Matthews L, Mitchell S, 2021. One health or three? Transmission modelling of *Klebsiella* isolates reveals ecological barriers to transmission between humans, animals and the environment. bioRxiv 10.1101/2021.08.05.455249

[GR276289HAWC51] Tong M, French S, Zahed SSE, Ong WK, Karp PD, Brown ED. 2020. Gene dispensability in *Escherichia coli* grown in thirty different carbon environments. mBio 11: e02259-20. 10.1128/mBio.02259-20 32994326 PMC7527729

[GR276289HAWC52] Tonkin-Hill G, MacAlasdair N, Ruis C, Weimann A, Horesh G, Lees JA, Gladstone RA, Lo S, Beaudoin C, Floto RA, 2020. Producing polished prokaryotic pangenomes with the Panaroo pipeline. Genome Biol 21: 180. 10.1186/s13059-020-02090-4 32698896 PMC7376924

[GR276289HAWC53] Vaser R, Šikić M. 2021. Time- and memory-efficient genome assembly with Raven. Nat Comput Sci 1: 332–336. 10.1038/s43588-021-00073-4 38217213

[GR276289HAWC54] Vezina B, Judd LM, McDougall FK, Boardman WSJ, Power ML, Hawkey J, Brisse S, Monk JM, Holt KE, Wyres KL. 2021. Transmission of *Klebsiella* strains and plasmids within and between Grey-headed flying fox colonies. bioRxiv 10.1101/2021.10.25.465810 PMC9790207 35590448

[GR276289HAWC55] Vimr ER, Troy FA. 1985. Identification of an inducible catabolic system for sialic acids (nan) in *Escherichia coli*. J Bacteriol 164: 845–853. 10.1128/jb.164.2.845-853.1985 3902799 PMC214328

[GR276289HAWC56] Walker BJ, Abeel T, Shea T, Priest M, Abouelliel A, Sakthikumar S, Cuomo CA, Zeng Q, Wortman J, Young SK, 2014. Pilon: an integrated tool for comprehensive microbial variant detection and genome assembly improvement. PLoS One 9: e112963. 10.1371/journal.pone.0112963 25409509 PMC4237348

[GR276289HAWC57] Wick RR, Judd LM, Gorrie CL, Holt KE. 2017. Unicycler: resolving bacterial genome assemblies from short and long sequencing reads. PLoS Comput Biol 13: e1005595. 10.1371/journal.pcbi.1005595 28594827 PMC5481147

[GR276289HAWC58] Wick RR, Judd LM, Cerdeira LT, Hawkey J, Méric G, Vezina B, Wyres KL, Holt KE. 2021. Trycycler: consensus long-read assemblies for bacterial genomes. Genome Biol 22: 266. 10.1186/s13059-021-02483-z 34521459 PMC8442456

[GR276289HAWC59] Wyres KL, Wick RR, Gorrie C, Jenney A, Follador R, Thomson NR, Holt KE. 2016. Identification of *Klebsiella* capsule synthesis loci from whole genome data. Microb Genom 2: e000102. 10.1099/mgen.0.000102 28348840 PMC5359410

[GR276289HAWC60] Wyres KL, Wick RR, Judd LM, Froumine R, Tokolyi A, Gorrie CL, Lam MMC, Duchêne S, Jenney A, Holt KE. 2019. Distinct evolutionary dynamics of horizontal gene transfer in drug resistant and virulent clones of *Klebsiella pneumoniae*. PLoS Genet 15: e1008114. 10.1371/journal.pgen.1008114 30986243 PMC6483277

[GR276289HAWC61] Wyres KL, Lam MMC, Holt KE. 2020. Population genomics of *Klebsiella pneumoniae*. Nat Rev Microbiol 18: 344–359. 10.1038/s41579-019-0315-1 32055025

[GR276289HAWC62] Yu G, Smith DK, Zhu H, Guan Y, Lam TT. 2017. ggtree: an R package for visualization and annotation of phylogenetic trees with their covariates and other associated data. Methods Ecol Evol 8: 28–36. 10.1111/2041-210X.12628

[GR276289HAWC63] Zhang Z, Aboulwafa M, Smith MH, Saier MH Jr. 2003. The ascorbate transporter of *Escherichia coli*. J Bacteriol 185: 2243–2250. 10.1128/JB.185.7.2243-2250.2003 12644495 PMC151508

[GR276289HAWC64] Zhu Y, Czauderna T, Zhao J, Klapperstueck M, Maifiah MHM, Han ML, Lu J, Sommer B, Velkov T, Lithgow T, 2018. Genome-scale metabolic modeling of responses to polymyxins in *Pseudomonas aeruginosa*. Gigascience 7: giy021. 10.1093/gigascience/giy021 PMC6333913 29688451

