# Supplementary material for: A curated collection of Klebsiella metabolic models reveals variable substrate usage and gene essentiality
Source: Genome Res. 2022 May;32(5):1004–14. doi: 10.1101/gr.276289.121 (PMC9104693; doi:10.1101/gr.276289.121)
Supplement: Supplemental Material [file supp_32_5_1004__DC1.html]

A curated collection of Klebsiella metabolic models reveals variable substrate usage and gene essentiality — Supplemental Material 

# A curated collection of *Klebsiella* metabolic models reveals variable substrate usage and gene essentiality

## Supplemental Material

- Supplemental\_Figure\_S1.pdf
- Supplemental\_Table\_S1.xlsx
- Supplemental\_Table\_S2.xlsx
- Supplemental\_Table\_S3.xlsx
- Supplemental\_Table\_S4.xlsx
- Supplemental\_Table\_S5.xlsx
- Supplemental\_Table\_S6.tsv.zip
- Supplemental\_Table\_S7.xlsx
- Supplemental\_Code.zip
