# Supplementary material for: A curated collection of Klebsiella metabolic models reveals variable substrate usage and gene essentiality
Source: Genome Res. 2022 May;32(5):1004–14. doi: 10.1101/gr.276289.121 (PMC9104693; doi:10.1101/gr.276289.121)
Supplement: Supplemental Material [file supp_gr.276289.121_Supplemental_Figure_S1.pdf]

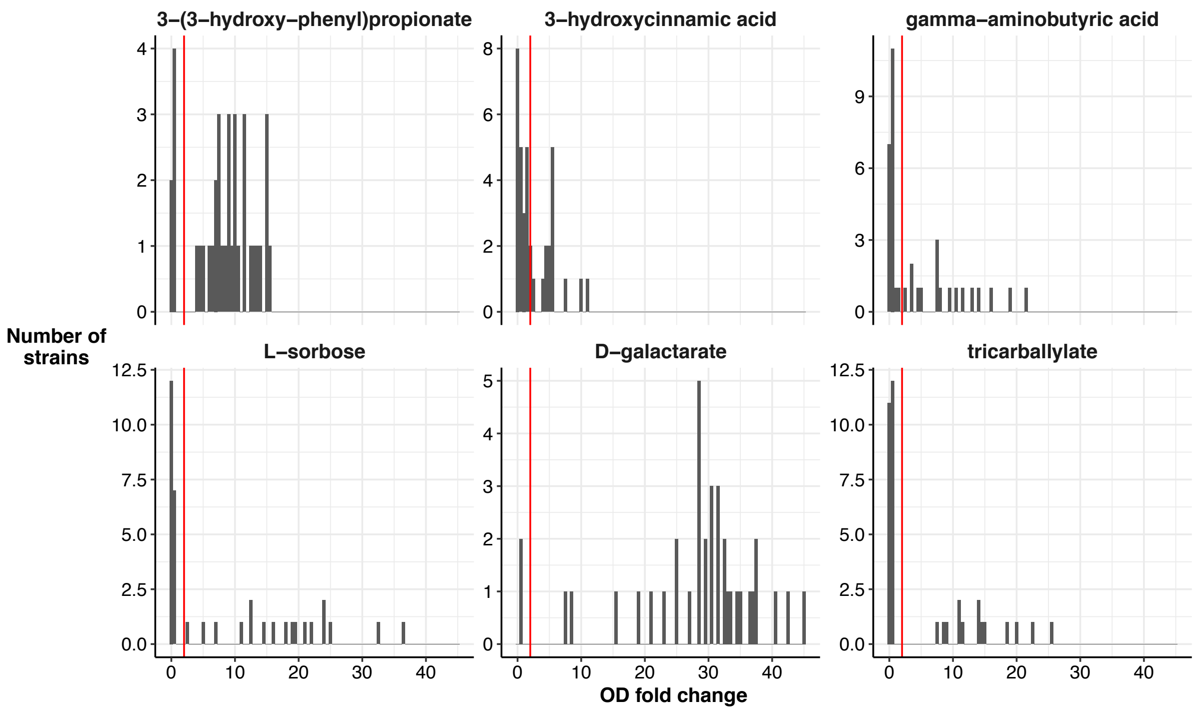

Supplemental Figure 1: Distribution of OD fold changes for growth on six carbon substrates. Each panel is a substrate showing total number of strains (y axis) with a particular OD fold change (x axis). Red line indicates OD fold change of 2, fold changes greater than this value were considered sufficient evidence of growth.
